# Association of the US Outbreak of Vaping-Associated Lung Injury With Perceived Harm of e-Cigarettes Compared With Cigarettes

**DOI:** 10.1001/jamanetworkopen.2020.6981

**Published:** 2020-06-15

**Authors:** Harry Tattan-Birch, Jamie Brown, Lion Shahab, Sarah E Jackson

**Affiliations:** 1 Institute of Epidemiology and Health Care, University College London, London, UK; 2 SPECTRUM Research Consortium, UK

## Abstract

This survey study assesses the perceptions of e-cigarettes among current smokers in England before and after the US outbreak of vaping-associated lung injury.

## Introduction

The US Food and Drug Administration acknowledges that nicotine and tobacco products exist on a continuum of risk, with e-cigarettes likely to be less harmful than combustible cigarettes because of their lower production of toxicants and carcinogens.^1,2^ However, many smokers in England and the US believe that e-cigarettes are at least as harmful to health as combustible cigarettes.^2,3^ These misperceptions likely dissuade smokers who are unable or unwilling to stop using nicotine from switching to e-cigarettes, which may have a detrimental effect on population health. The recent US outbreak of vaping-associated lung injury (EVALI)^4^ received extended news coverage worldwide. Most cases were associated with inhalation of vitamin E acetate, an additive found in some tetrahydrocannabinol vaping devices.^5^ However, news reports often failed to distinguish tetrahydrocannabinol devices from standard nicotine-based e-cigarettes, which may have increased confusion about the relative harms of different nicotine products. This study examined the extent to which perceptions of the harm of e-cigarettes compared with combustible cigarettes changed among smokers after the EVALI outbreak.

## Methods

This survey study used data from the Smoking Toolkit Study, a monthly cross-sectional nationally representative survey of adults (aged >16 years) in England. Oral informed consent was obtained from participants, and ethical approval was granted by the University College London Ethics Committee. The study followed American Association for Public Opinion Research (AAPOR) reporting guideline.

Current smokers were asked, “Compared to regular cigarettes, do you think electronic cigarettes are more, less, or equally harmful to health?” They could also respond, “don’t know.” Self-reported sex, age, socioeconomic status, race/ethnicity, and current e-cigarette use were also measured. The analysis plan was preregistered.

The majority of EVALI hospitalizations were between mid-August and mid-September 2019,^4^ and internet searches for *vaping* and *vaping death* peaked mid-September.^6^ Thus, we compared harm perceptions in 2019 before the EVALI outbreak (January to July 2019) with those after the outbreak (August to December 2019). Log-binomial regression was used to assess the association between timing of the outbreak and the proportion of smokers who believed that e-cigarettes were less harmful than cigarettes before and after adjusting for sociodemographic factors and e-cigarette use. In secondary analyses, we calculated associations between timing of the outbreak and the proportion of people reporting each of the other responses. Analyses were conducted using R, version 3.5.3 (R Foundation for Statistical Computing). A 1-sided *P* < .05 was considered to be statistically significant.

## Results

Of the 3215 current smokers surveyed in 2019, 1833 were interviewed before the outbreak (849 [46.3%] women; mean [SD] age, 43.5 [17.6] years) and 1382 were interviewed after (604 [43.7%] women; mean [SD] age, 43.0 [17.8] years). The proportion who perceived e-cigarettes as less harmful than combustible cigarettes decreased significantly from 37.0% (n = 678) before to 30.9% (n = 427) after the outbreak (risk ratio [RR], 0.83; 95% CI, 0.76-0.92; *P* < .001), and significantly fewer smokers reported not knowing which product was more harmful (191 [10.4%] vs 112 [8.1%]; RR, 0.78; 95% CI, 0.63-0.98; *P* = .03). Conversely, there were significant increases in the proportion of individuals who perceived e-cigarettes as equally harmful (731 [39.9%] vs 605 [43.8%]; RR, 1.10; 95% CI, 1.01-1.19; *P* = .01) or more harmful (233 [12.7%] vs 238 [17.2%]; RR, 1.36; 95% CI, 1.15-1.61; *P* < .001) than cigarettes. All significant differences remained after adjustment for covariates (Table).

**Table.** Harm Perceptions of e-Cigarettes Compared With Cigarettes Among Current Smokers in England Before (January to July 2019) and After (August to December 2019) the Outbreak of Vaping-Associated Lung Injury

Harm perception	Smokers, No. (%) [95% CI]		Unadjusted risk ratio (95%CI)	*P* value	Adjusted risk ratio (95%CI)^a^	*P* value
	Before outbreak (n = 1833)	After outbreak (n = 1382)				
Less harmful	678 (37.0) [34.7-39.3]	427 (30.9) [28.5-33.4]	0.83 (0.76-0.92)	<.001	0.81 (0.74-0.90)	<.001
Equally harmful	731 (39.9) [37.7-42.2]	605 (43.8) [41.2-46.4]	1.10 (1.01-1.19)	.01	1.09 (1.01-1.18)	.02
More harmful	233 (12.7) [11.2-14.3]	238 (17.2) [15.3-19.3]	1.36 (1.15-1.61)	<.001	1.38 (1.17-1.62)	<.001
Don’t know	191 (10.4) [9.1-11.8]	112 (8.1) [6.8-9.7]	0.78 (0.63-0.98)	.03	0.78 (0.62-0.97)	.03

^a^ Adjusted analyses included sex, age, social grade, race/ethnicity, and e-cigarette use as covariates.

The Figure shows harm perceptions among smokers from 2016 through 2019. In the final quarter of 2019, the percentage of individuals who perceived e-cigarette use as less harmful than cigarette smoking decreased to the lowest point recorded (239 [29.5%]; 95% CI, 26.5%-32.8%), and the percentage perceiving it as more harmful peaked (155 [19.2%]; 95% CI, 16.6%-22.0%).

**Figure.**
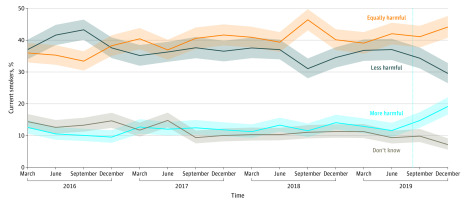
Perceived Harm of e-Cigarettes Compared With Cigarettes Among Smokers in England From 2016 to 2019 A total of 13 781 current smokers were surveyed (approximately 860 per quarter). Solid lines represent means; shaded areas, 95% CIs; and dotted vertical line, the peak of the vaping-associated lung injury outbreak.

## Discussion

After the US outbreak of vaping-associated lung injury, views on e-cigarettes among smokers in England deteriorated. The proportion perceiving e-cigarette use as less harmful than cigarette smoking decreased, and the proportion perceiving e-cigarette use as more harmful increased by over one-third.

The effects that these worsened harm perceptions will have on population health is unclear. It is possible that people who had quit smoking cigarettes through vaping might now return to smoking, and cigarette smokers might be deterred from using e-cigarette devices to help them quit. On the other hand, young people who have never smoked may be dissuaded from ever trying e-cigarettes. A limitation of this study is that only smokers in England were surveyed. The association between EVALI and harm perceptions may differ across countries. In the US, where the outbreak occurred and precipitated fierce political debate, there may have been an even greater change. These results highlight the importance of clear communication from public health bodies about the relative harm of different nicotine products. Future research should explore how these changing perceptions affect smoking and e-cigarette use prevalence in the US and beyond.

## References

[1] US Food and Drug Administration FDA announces comprehensive regulatory plan to shift trajectory of tobacco-related disease, death. 2018 Accessed November 22, 2019. https://www.fda.gov/news-events/press-announcements/fda-announces-comprehensive-regulatory-plan-shift-trajectory-tobacco-related-disease-death

[2] Mcneill A, Brose LS, Calder R, Bauld L, Robson D Evidence review of e-cigarettes and heated tobacco products 2018: a report commissioned by Public Health England. Public Health England; 2018.

[3] Nyman AL, Huang J, Weaver SR, Eriksen MP Perceived comparative harm of cigarettes and electronic nicotine delivery systems. JAMA Netw Open. 2019;2(11):e1915680. doi:10.1001/jamanetworkopen.2019.15680 31747029 PMC6902805

[4] Centers for Disease Control and Prevention Outbreak of lung injury associated with the use of e-cigarette, or vaping, products. 2019 Accessed November 22, 2019. https://www.cdc.gov/tobacco/basic_information/e-cigarettes/severe-lung-disease.html#latest-outbreak-information

[5] Nyakutsikwa B, Britton J, Bogdanovica I, Langley T Vitamin E acetate is not present in licit e-cigarette products available on the UK market. *Addiction* January 2020:add.14920. doi:10.1111/add.14920 31785207

[6] Google Interest in “vaping” and “vaping deaths” from 2018 to 2019. Google Trends. Published 2019 Accessed April 30, 2020. https://trends.google.com/trends/explore/TIMESERIES/1574436000?hl=en-GB&tz=0&geo=GB&q=vaping,vaping+deaths&sni=3

